# Selection and Validation of Reference Genes for Quantitative Real-Time PCR Analysis of Development and Tissue-Dependent Flower Color Formation in *Cymbidium lowianum*

**DOI:** 10.3390/ijms23020738

**Published:** 2022-01-10

**Authors:** Xiu-Mei Dong, Wei Zhang, Shi-Bao Zhang

**Affiliations:** 1 Key Laboratory of Economic Plants and Biotechnology, Kunming Institute of Botany, Chinese Academy of Sciences, 132# Lanhei Road, Kunming 650201, China; dongxiumei@mail.kib.ac.cn (X.-M.D.); zhangwei@mail.kib.ac.cn (W.Z.); 2 Yunnan Key Laboratory for Wild Plant Resources, 132# Lanhei Road, Kunming 650201, China

**Keywords:** *Cymbidium lowianum*, flower organ development, reference gene selection and validation, qRT-PCR, transcriptome data, geNorm and Normfinder software

## Abstract

The development and tissue-dependent color formation of the horticultural plant results in various color pattern flowers. Anthocyanins and carotenoids contribute to the red and yellow colors, respectively. In this study, quantitative real-time polymerase chain reaction (qRT-PCR) is used to analyze the expression profiles of anthocyanin and carotenoids biosynthesis genes in *Cymbidium lowianum* (Rchb.f.) Rchb.f. Appropriate reference gene selection and validation are required before normalization of gene expression in qRT-PCR analysis. Thus, we firstly selected 12 candidate reference genes from transcriptome data, and used geNorm and Normfinder to evaluate their expression stability in lip (divided into abaxial and adaxial), petal, and sepal of the bud and flower of *C. lowianum*. Our results show that the two most stable reference genes in different tissues of *C. lowianum* bud and flower are *EF1δ* and *60S*, the most unstable reference gene is *26S*. The expression profiles of the *CHS* and *BCH* genes were similar to FPKM value profiles after normalization to the two most stable reference genes, *EF1δ* and *60S*, with the upregulated *CHS* and *BCH* expression in flower stage, indicating that the ABP and CBP were activated across the stages of flower development. However, when the most unstable reference gene, *26S*, was used to normalize the qRT-PCR data, the expression profiles of *CHS* and *BCH* differed from FPKM value profiles, indicating the necessity of selecting stable reference genes. Moreover, *CHS* and *BCH* expression was highest in the abaxial lip and adaxial lip, respectively, indicating that the ABP and CBP were activated in abaxial and adaxial lip, respectively, resulting in a presence of red or yellow segments in abaxial and adaxial lip. This study is the first to provide reference genes in *C. lowianum*, and also provide useful information for studies that aim to understand the molecular mechanisms of flower color formation in *C. lowianum*.

## 1. Introduction

**Figure 1:**
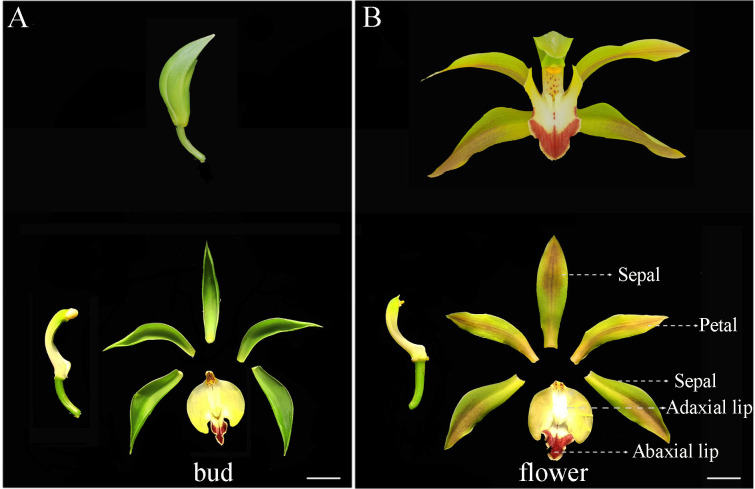
Two stages of *C. lowianum* flower development: (**A**) bud and (**B**) flower. V-shaped red abaxial, and adaxial of lip, petal, and sepal of bud and flower were collected as samples for this study. Bar = 0.5 cm.

Flower color is a pivotal plant phenotype for ornamentation and population diversity [1,2]. Accordingly, investigating the formation of flower color is key to understand the evolution of color variation in plant populations and may facilitate ornamental plant breeding [3,4]. *Cymbidium lowianum* (Rchb.f.) Rchb.f. (Orchidaceae) is an important ornamental plant that possess flowers with a deep red V-shape on the abaxial lip in flower stage [5,6]. Moreover, in the bud stage, the color of sepal and petal are green (Figure 1A). In the flower stage, sepal and petal turned yellow green, and reddish brown on some longitudinal veins (Figure 1B). However, the molecular mechanisms of flower color formation in *C. lowianum* remains unclear. Gene expression analysis is fundamental to understanding gene function and the molecular mechanisms of developmental processes [7].

Quantitative real-time polymerase chain reaction (qRT-PCR) is one of the most common and important tools in the detection of gene expression due to its high sensitivity, accuracy and reliability [8]. The reliability and accuracy of qRT-PCR largely depend on the stability of reference genes, and the specificity of reference gene primer pairs [9]. Thus, the selection of appropriate reference genes and design of specific primers are prerequisites for ensuring the accuracy of qRT-PCR results [10]. Housekeeping genes are often used as standardized reference genes, but the expression of many housekeeping genes varies greatly in different tissues, different species, and under different environmental conditions [7,11,12,13,14,15,16].

To date, qRT-PCR reference genes have been identified in many plants, and have been used to analyze changes in gene expression during development and in response to environmental stimuli. For example, over the past 10 years, stable reference genes for plant developmental analysis have been identified in *Agaricus blazei* [15], *Euscaphis konishii* [17], *Gentiana macrophylla* [18], *chrysanthemums* [12,13], *Chinese wolfberry* [19], *Solanum melongena L* [20], maize [21], and *Eremosparton songoricum* [22]. Similarly, stable reference genes have been identified to analyze changes in gene expression changes in response to abiotic and biotic stresses in *Suaeda glauca* [16], maize [23], *Isatis indigotica* [24], *Lolium multiflorum* [25], *Brassica napus* [26], and lettuce [27]. Stable reference genes have yet to be reported for orchids, including *C. lowianum*. Furthermore, these studies indicate that stable reference genes may differ in plant species and when examining different abiotic stresses.

Plant genome and transcriptome sequences have been used to select candidate reference genes in a wide variety of plants, including *Agaricus blazei* [15], *Euscaphis konishii* [24], *Stellera chamaejasme* [28], *Lolium perenne* [29], *Arabidopsis thaliana* [30], and *Solanum lycopersicum* [31]. The *C. lowianum* genome has yet to be sequenced, although the chloroplast genome has recently been sequenced. Furthermore, morphological and phylogenetic analyses revealed a close relationship between *C. lowianum* and *C. tracyanum* [6]. Our team has sequenced and assembled the genome of *C. tracyanum* (unpublished), and found that reads of *C. lowianum* transcriptome data could map 85.76–90.94% to the genome of *C. tracyanum,* indicating a close relationship between them at the whole genome level. These findings suggest it is feasible to select reference genes from *C. lowianum* RNA sequencing data.

In this study, we selected 12 candidate reference genes (*ACT7*, *ACTF11*, *ATP*, *EF1δ*, *EIF*, *MADH*, *RAD23d*, *g-TUB*, *UBC*, *26S*, *40S*, and *60S*) according to their expression in *C. lowianum* transcriptome data. We then used geNorm [32] and NormFinder [33] to validate the appropriateness of these candidate reference genes for qRT-PCR analysis of gene expression. Finally, we detected the expression profiles of chalcone synthase (*CHS*) and β-carotene hydroxylase (*BCH*), structural genes of anthocyanin and carotenoid biosynthesis pathways (ABP and CBP) [34,35] in different flower tissues across the stages of flower development in *C. lowianum*.

## 2. Results

### 2.1. Selection of Candidate Reference Genes Based on Transcriptome Data

To obtain appropriate reference genes in *C.* *lowianum,* we firstly searched transcriptome data for genes without significant difference in expression in tissues (abaxial lip, adaxial lip, petal, and sepal) at two stages of flower development (bud and flower) (Figure 1). In order to obtain accuracy results in qRT-PCR analysis, the Cq value of reference genes should range from 20 to 30 [36]. Based on our experience, when the FPKM values of genes are about 100 in the samples, the Cq values range from 20 to 30 in qRT-PCR analysis following the protocol in the present methods. Thus, we searched *C. lowianum* transcriptome data for genes that showed FPKM values of ~100, and identified 10 candidate reference genes. The common housekeeping genes, *ACT* and *TUB*, were not among these candidates; a subsequent search for *ACT* and *TUB* in transcriptome data identified *ACT7* and *g-TUB*, which had the most stable expression among the homologues of *ACT* and *TUB*. Thus, the total number of candidate reference genes for further study was 12 (Table S1).

### 2.2. Primer Specificity and Amplification Efficiency Test of Candidate Reference Genes

The specificity and amplification efficiency of primer sets for each candidate reference genes were tested by RT-PCR and qRT-PCR analysis. The gene names, descriptions, primer sequences, amplicon length, amplification efficiencies, and R^2^ are given in Table 1. In RT-PCR analysis, only 10 candidate reference genes showed a single band in 2.0% agarose gel; *EIF* and *RAD23d* had no band in the agarose gel (Figure S1). Thus, we excluded *EIF* and *RAD23d* from further qRT-PCR analyses. The specificity for each primer set was also validated by the melting curves of qRT-PCR. For all primer sets, the melting curve showed a single amplification peak (Figure S2), indicating that the primers were highly specific.

For amplification efficiency analysis, five gradients (1, 1/5, 1/25, 1/125, and 1/625) of mixed cDNA of all samples were used for qRT-PCR to produce standard curves, and calculate the amplification efficiency (*E*) (*E* = 10^−1/k^ − 1). The abscissa of the standard curves are log_10_ (1, 1/5, 1/25, 1/125, and 1/625), the ordinate of the standard curves were the corresponding cycle threshold (Cq) values, k indicates slope factor of the standard curves, R^2^ indicates correlation coefficients (Figure S3). As shown in Table 1, the amplification efficiency of qRT-PCR for all 10 candidate reference genes ranged from 88.58% (*ACT7*) to 158.30 (*ACTF11*), and R^2^ ranged from 0.8844 (*ACTF11*) to 0.9999 (*40S*).

### 2.3. Expression Profiles of Candidate Reference Genes

**Figure 2:**
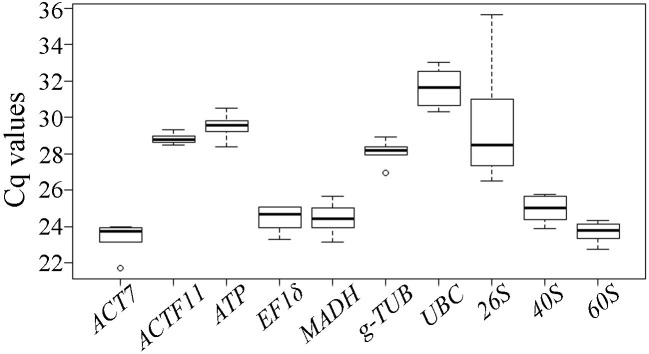
qRT-PCR cycle threshold (Cq) values of 10 candidate reference genes from all investigated samples. The lines across the box indicate the median values, boxes indicate the 25/75 percentiles, whisker caps indicate the maximum and minimum values, and small circles represent outliers. The higher boxes and whiskers indicate the greater the variations.

The expression profiles are represented by Cq values of the candidate reference genes in different samples. The Cq values of the 10 candidate reference genes were detected by qRT-PCR. As shown in Figure 2, the expression levels across all investigated samples showed variation among the candidate reference genes. The Cq values of the 10 candidate reference genes displayed a wide range from 21.70 (*ACT7*) to 33.03 (*UBC*) in all samples, and mean Cq ranged from 23.42 (*ACT7*) to 31.62 (*UBC*), indicating that *ACT7* had the highest expression level, whereas *UBC* had the lowest expression level in the samples. In addition, *26S* expression levels were the most variable with 9.17 Cq; *ACTF11* showed the least variable levels with 0.84 Cq.

### 2.4. Expression Stability of Candidate Reference Genes

After the above qRT-PCR assays, we used geNorm and NormFinder to evaluate the expression stability of the 10 candidate reference genes. To identify reference genes that can be used to examine gene expression changes in diverse tissues, we evaluated the stability of candidate reference gene expression in several tissues at the same stage of development, i.e., in the abaxial lip, adaxial lip, petal, and sepal of the bud; and in the abaxial lip, adaxial lip, petal, and sepal of the flower (tissue-dependent group). To identify reference genes that can be used to examine gene expression changes in whole flower across developmental stages, we evaluated the stability of candidate reference gene expression in the bud and flowering stages. To identify reference genes that can be used to examine gene expression changes in a single tissue across developmental stages, we evaluated the stability of candidate reference gene expression in each individual tissue across developmental stages, i.e., abaxial lip of bud and flower; adaxial lip of bud and flower petal of bud and flower; sepal of bud and flower; and all tissues of bud and flower (development-dependent group).

#### 2.4.1. geNorm Analysis

**Figure 3:**
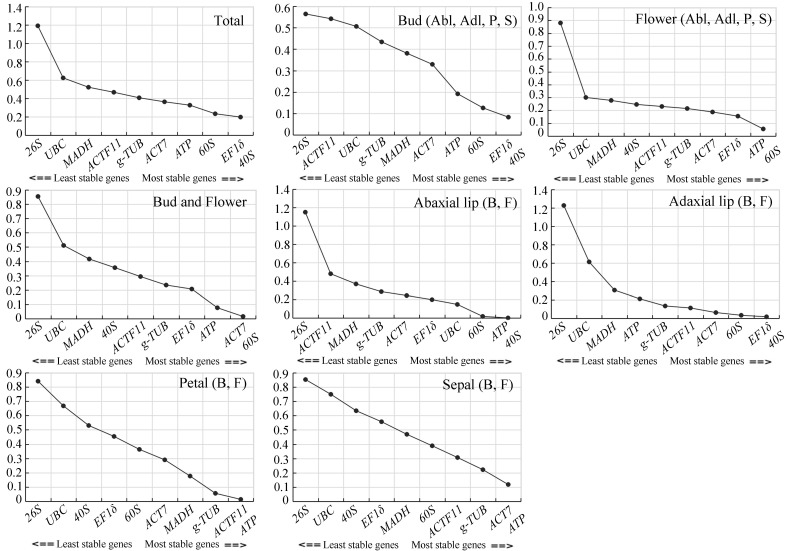
Average expression stability (M value) of 10 candidate reference genes calculated by geNorm software. Expression stability was evaluated in different sample combinations. Totals indicate samples of abaxial lip, adaxial lip, petal, and sepal from the bud and flower. Abl, Adl, P, and S indicate abaxial lip, adaxial lip, petal, and sepal, respectively. Bud and flower indicate samples of two developmental stages. B and F indicate bud and flower, respectively. The lower M value, the more stable expression of the reference genes.

**Figure 4:**
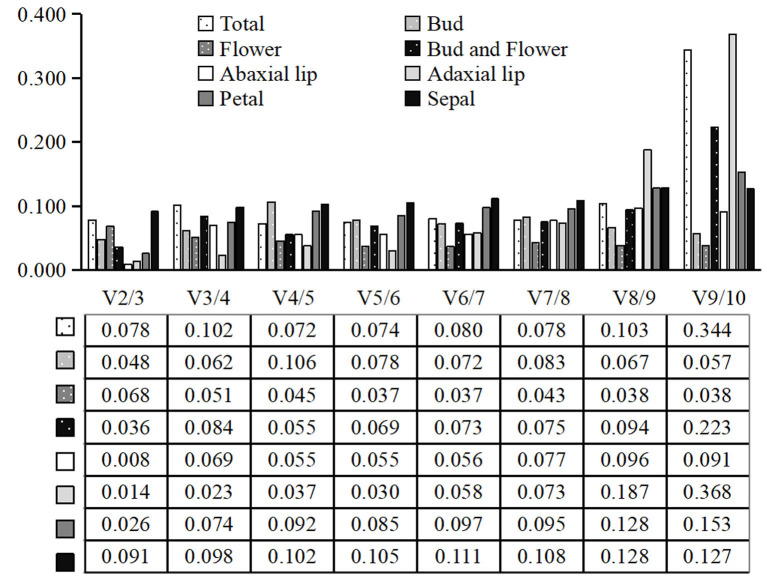
Optimal number of reference genes in different groups calculated by geNorm. The pairwise variation (Vn/Vn + 1) was analyzed between normalization factors (NFn and NFn + 1). The Vn/n + 1 threshold is 0.15. If Vn/n + 1 is lower than 0.15, n reference genes can meet the experimental requirements, otherwise n + 1 reference genes should be used for further study.

The gene expression values were evaluated by M value in geNorm analysis, with the default limit value of 1.5, lower M value indicated greater gene expression stability [5]. The results of geNorm analyses are shown in Figure 3. The three genes with the most stable expression across multiple tissues and developmental stages were *60S*, *EF1δ*, and *40S*. These three genes also had the most stable expression in the bud stage; however, in the flowering stage, the genes with the most stable expression were *EF1δ*, *ATP*, and *60S*. The candidate reference genes with the most stable expression differed in different tissues across developmental stages. For instance, in the whole bud and flower, the three genes with the most stable expression were *ATP*, *ACT7*, and *60S*. In the abaxial lip, it was *60S*, *ATP*, and *40S*; in the adaxial lip, *60S*, *EF1δ*, and *40S*; in the petal, *g-TUB*, *ACTF11*, and *ATP*; and in the sepal, *g-TUB*, *ACT7*, and *ATP*. We also used geNorm to calculate the optimal number of reference genes required for normalization in different groups. The V2/3 values for all the different groups were below the threshold 0.15, indicating that two reference genes were enough to normalize qRT-PCR data (Figure 4). Therefore, combining the M values and Vn/n+1 values given by geNorm, we found that *EF1δ/40S* and *60S* were the most stable genes amongst all samples, *EF1δ/40S* and *60S* were the most stable genes in all tissues of the bud stage, and *ATP*/*60S* and *EF1δ* were the most stable genes in all tissues of the flower stage. Together, in the tissue-dependent group, the most stable genes were *EF1δ* and *60S*. In the development-dependent group, *ACT7*/*60S* and *ATP* were the most stable genes in the whole bud and flower; *ATP*/*40S* and *60S*, *EF1δ/40S* and *60S*, *ACTF11*/*ATP* and *g-TUB*, and *ACT7*/*ATP* and *g-TUB* were the most stably expressed combinations in abaxial lip, adaxial lip, petal, and sepal across flower developmental stages, indicating that the most stable genes differed in abaxial lip, adaxial lip, petal, and sepal. The least stable reference gene in all groups was *26S*.

#### 2.4.2. NormFinder Analysis

In NormFinder analysis, a direct variation value was used to evaluate the stability of the reference genes. Lower variation values indicate more stability of the reference genes; otherwise, more instability of the reference genes. In the tissue-dependent group, *EF1δ, 60S*, and *40S* were the most stable genes amongst all samples; *60S*, *EF1δ*, and *ATP* were the most stable genes in all tissues of the bud stage; *ATP*, *60S*, and *EF1δ* were the most stable genes in all tissues of flower stage. In the development-dependent group, *ACT7*, *60S,* and *EF1δ* were the most stable genes in the whole bud and flower, petal, and sepal; *ATP*, *40S,* and *60S* were the most stable genes in abaxial lip across the flower developmental stages; *ACTF11*, *g-TUB* and *60S* were the most stably expressed combinations in adaxial lip across the flower developmental stages. The least stable reference gene in all groups was *26S* as well (Table 2).

### 2.5. The Expression Profiles of CHS and BCH Validated by the Most Stable/unstable Reference Genes

**Figure 5:**
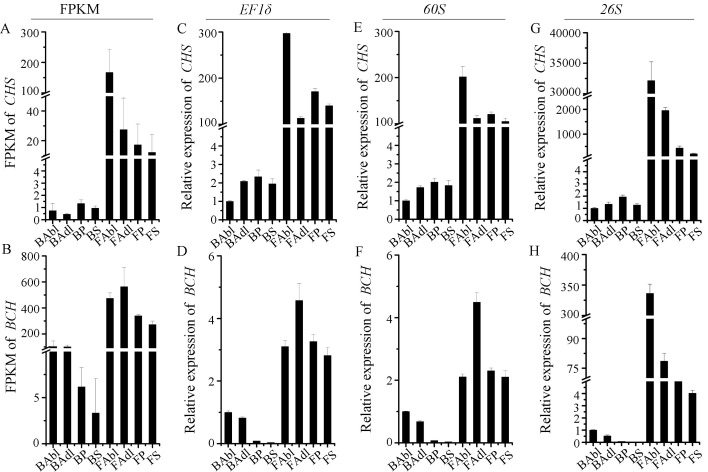
The expression profiles of *CHS* and *BCH* in *C. lowianum.* (**A**,**B**) The expression levels of *CHS* and *BCH* presented by FPKM value of transcriptome data. (**C**–**F**) The expression levels of *CHS* and *BCH* normalized by the most stable reference genes *EF1δ* (**C**,**D**), and *60S* (**E**,**F**). (**G**,**H**) The expression levels of *CHS* and *BCH* normalized by the most unstable reference genes *26S.* BAbl, BAdl, BP, and BS indicate abaxial lip, adaxial lip, petal, and sepal of the bud, respectively. FAbl, FAdl, FP, and FS indicate abaxial lip, adaxial lip, petal, and sepal of the flower, respectively.

Anthocyanins and carotenoids are widely distributed pigments in flowers, and their biosynthetic pathways (ABP and CBP) have been extensively studied in model and ornamental plants [3,37]. Chalcone synthase (*CHS*) and β-carotene hydroxylase (*BCH*) encode structural proteins that play roles in anthocyanin and carotenoid biosynthesis, respectively. The expression profiles of *CHS* and *BCH* were firstly showed by FPKM values in transcriptome data (Figure 5A,B). To validate the transcriptome data and the appropriateness of the reference genes, the two most stable reference genes, *EF1δ* (Figure 5C,D) and *60S* (Figure 5E,F), and the least stable reference gene *26S* (Figure 5G,H) were used to analyze the expression profiles of *CHS* and *BCH* in different tissues (abaxial lip, adaxial lip, petal, and sepal) across the flower developmental stages (bud and flower). When *EF1δ* and *60S* were used to normalize expression, *CHS* and *BCH* expression patterns were similar to those shown by their respective FPKM value profiles, with the upregulated *CHS* and *BCH* expression in flower tissue, indicating that the ABP and CBP were activated across the stages of flower development. In addition, *CHS* expression was highest in the abaxial lip (Figure 5A,C,E), and *BCH* expression was highest in the adaxial lip (Figure 5B,D,F), indicating that the ABP and CBP were activated in abaxial and adaxial lip, resulting in a presence of red or yellow segments in abaxial and adaxial lip, respectively. When *26S* (unstable gene) was used for normalization, the relative expression patterns of *CHS* and *BCH* differed from the relative expression patterns obtained using the two most stable reference genes (*EF1δ* and *60S*) (Figure 5G,H). These results confirmed the importance of selecting reference genes prior to qRT-PCR analysis.

## 3. Discussion

In this study, we identified 12 candidate reference genes from transcriptome data of *C. lowianum* and analyzed their stability. We used geNorm and NormFinder to calculate expression stability of 10 candidate reference genes. In addition, qRT-PCR was used to detect the expression profiles of *CHS* and *BCH* in different samples across the stages of *C. lowianum* flower development, and validate the appropriateness of the results of geNorm and NormFinder analysis.

With the rapid development of high-throughput sequencing technology, RNA-sequencing has been widely used to detect gene expressions, based on genome sequence. The Orchidaceae is one of the largest families of flowering plants. However, due to the difficulty of assembling and annotating of genomes of *Cymbidium*, only *Cymbidium goeringii* [38] and *Cymbidium sinense* [39] had been sequenced and published. Morphological and phylogenetic analyses revealed a close relationship between *C. lowianum* and *C. tracyanum* [6]. Moreover, the reads of our *C. lowianum* transcriptome data could map 85.76–90.94% to the genome of *C. tracyanum,* which also indicate a close relationship between them at the whole genome level. In addition, transcriptome data of *C. lowianum* could be used to analyze gene expressions, and presumptive function genes associated with flower color formation in different tissues across the stages of flower development.

In the tissue-development group, our results show that the two most stable reference genes in different tissues of *C. lowianum* bud and flower are *EF1δ*, and *60S*. These results coincide with the previous studies that *60S* is the most suitable reference gene in different tissues in soybean [40,41], and in five developmental stages of stems in *Panax ginseng* [42]. Moreover, *60S* is also found to be the most stable reference gene under various stress conditions [41]. However, there are few studies that have chosen *EF1δ* as a reference gene in plants, but *EF1α* was found to be the most suitable reference gene in different tissues in *Panax ginseng* [42] and jute [43]. *ACT7* and g-*TUB*, two of the most widely used reference genes, showed different expression stabilities. In this study, *ACT7* did not show a good expression stability in abaxial lip, adaxial lip, and petal, while they showed a good expression stability in sepal during *C. lowianum* flower development from the bud to flower. g-*TUB* did not show a good expression stability in all tissues during *C. lowianum* flower development from the bud to flower. In the previous studies, there are also few studies that have chosen *g-TUB* as a reference gene in plants, but *β-TUB* and *α-TUB* were used as reference genes usually, which did not among the selected candidate reference genes in this study. *α-TUB* is the least suitable reference gene in different tissues in *Platycladus orientalis* [44], and *β-TUB* exhibited low stability in *Glehnia littoralis* [45], while it showed the most stability in Jute [43]. *ACT7* also showed different expression stabilities in different experimental conditions. *ACT7* showed the least stable expression patterns during fruit ripening of red pitaya [46], in the seedlings of *Suaeda glauca* under NaCl treatment [16], and in different flax tissues [47]; while it showed the most stable expression patterns across different tissues and cold-treated samples in *Platycladus orientalis* [44], and in flixweed (*Descurainia sophia*) [48]. In the development-dependent group, the two most stable genes in the different tissues (abaxial lip, adaxial lip, petal, and sepal) differed (Figure 3, Table 2), coincided with the results in *Panax ginseng* [42]. These differences in reference gene expression across developmental stages and/or individual tissues demonstrate that gene expression is not always stable for a single given reference gene. In *C. lavandulifolium*, *SAND* (SAND family protein) is the most stable reference gene in different development samples and different tissues during the process of flower development [13], indicating that no single reference gene can be used for all species or different experimental conditions [13,49].

GeNorm and NormFinder are the two most widely used software for evaluating reference genes. In this study, the most stable reference genes identified by using GeNorm and NormFinder. In the tissue-dependent group, both GeNorm and NormFinder indicated that the two most stable reference genes are *EF1δ* and *60S*. In the development-dependent group, combined GeNorm and NormFinder results showed the most stable reference genes in abaxial lip to be *ATP* and *40S*, in adaxial lip to be *60S* and *EF1δ*, in petal and sepal to be *ACT7* and *ATP* (Figure 3, Table 2). Taken together, these findings indicate that the most stable reference gene for expression analysis during *C. lowianum* flower development differed in different experimental conditions.

The expression levels of structural genes in the pigment of anthocyanins and carotenoids biosynthesis pathways can directly influence pigment accumulation to determine flower color [37,50,51]. However, little is known about the molecular mechanisms of color formation in lip (divided into abaxial and adaxial), petal, and sepal across *C. lowianum* flower development stages. The expression profiles of *CHS* and *BCH* in *C. lowianum*, indicate that both anthocyanins and carotenoids content may increase from the bud stage to the flower stage. In the bud stage, ABP may be activated in lip, sepal, and petal, while BCP may be activated in lip, and inactivated in sepal and petal. In the flower stage, ABP and BCP may be both activated in lip, sepal, and petal. Moreover, in the abaxial lip of the flower, ABP may be mainly activated, resulting in the red segment; in the adaxial lip of flower, BCP may be mainly activated, resulting in the yellow segment.

This study is the first to select reference genes and detect anthocyanins and carotenoids biosynthesis pathway structural gene (*CHS* and *BCH*) expression profiles in different tissues across the stages of flower development in *C.* *lowianum*. Our findings provide a foundation for gene expression analysis during *C. lowianum* flower development and research aimed at elucidating the mechanisms of flower color formation. Moreover, these results confirm that different reference genes should be used for normalizing qRT-PCR data under different experimental conditions.

## 4. Materials and Methods

### 4.1. Plant Materials

*C.lowianum* was cultivated in a greenhouse at Kunming Institute of Botany, Chinese Academy of Sciences greenhouse (Kunming, China) under natural light at a temperature between 24–28 °C. *C. lowianum* flower tissues (abaxial lip, adaxial lip, petal, and sepal) were collected at the bud stage and at 10 days after flowering. All samples were immediately frozen in liquid nitrogen and then stored at −80 °C.

### 4.2. RNA Preparation, Reverse Transcription and qRT-PCR

Total RNA was extracted from tissues using RNAprep Pure Plant kit (TIAGEN, Beijing, China). RNA amounts were estimated using a NanoDrop 2000 (Thermo Fisher Scientific, Waltham, MA USA). cDNA synthesis was performed with approximately 2 μg of total RNA and the reaction system followed the PrimeScript^TM^ RT reagent Kit with gDNA Eraser (TaKaRa, RR047A, Shiga, Japan) protocol. The cDNA was eluted with 100 μL deionized water. A total of 2 μL of eluted cDNA was used as a template for qRT-PCR analysis. qRT-PCR was performed on a BioRad sequence detection system using TB Green Premix Ex Taq II (Tli RNaseH Plus) (TaKaRa, RR820, Shiga, Japan) for detection. Amplification conditions were as follows: 3 min of initial denaturation at 95 °C, followed by 40 cycles of 10 s at 95 °C, 20 s at 55 °C, and 20 s at 72 °C. Melt curves used to determine primer specificity were 10 s at 95 °C, followed by cycles of 0.05 s at 65–95 °C, rising 0.5 °C/cycle. Primers are listed in Table 1.

### 4.3. Screening of Candidate Reference Genes

In the transcriptome data sets, gene expression levers were calculated by FPKM values [52]. Genes without significant difference in FPKM values among the samples were selected as candidate reference genes. From these genes, 10 candidate reference genes (*ACTF11*, *ATP*, *EF1δ*, *EIF*, *MADH*, *RAD23d*, *UBC*, *26S*, *40S*, and *60S)* with the FPKM values of about 100, and two common housekeeping genes, *ACT7* and *g-TUB*, with the most stable expression among their homologous genes, were chosen for further study.

### 4.4. Primer Design and qRT-PCR Analysis of Candidate Reference Genes

Primers were selected artificially, and then tested using DNAMAN software with the following criteria: primer lengths between 18 to 25 bp, melting temperature between 50 to 60 °C, GC content between 40 to 60%, and amplicon lengths between 100 to 200 bp (Table 1). The expression level of each candidate gene was evaluated based on qRT-PCR. The specificity of the candidate reference genes was verified by RT-PCR, the products of which were visualized with 2% agarose gel electrophoresis (Figure S1), and qRT-PCR, as represented by a melting curve (Figure S2). The amplification efficiency (E) of each candidate gene was calculated according to a previous study [53,54].

### 4.5. Selection of Reference Genes with Stable Expression

To evaluate the stability of gene expression for each candidate reference gene, we used geNorm [32] and NormFinder [33] according to a recent study [17]. Before using this software, the minimum Cq values of candidate genes in all samples was determined, ∆Ct = Cq values of the other samples subtracting the minimum Cq among the samples, relative quantities were calculated by 2^−^^∆Ct^. The values that represent the relative expression of each candidate reference gene in different samples were exported into an Excel datasheet (Microsoft Excel 2003), and the Ct values were converted according to the requirements of the software. In geNorm analysis, M values represent the pairwise variation for the candidate reference genes; using a cut-off of 1.5, lower M values indicate stable gene expression [32]. The geNorm software can also calculate a normalization factor for each experimental condition and suggest an optimal number of reference genes necessary for the experiment. In NormFinder analysis, a direct variation value of the candidate reference genes is provided [33]. Each of these approaches generates a rank order of the reference genes that represent reference gene stability. Based on the evaluated results of the two software, the most stable reference genes for qRT-PCR in different experimental conditions of *C.* *lowianum* were selected.

### 4.6. Validation of the Candidate Reference Genes

To confirm the reliability of the potential reference genes, we measured and normalized the relative expression of *CHS* and *BCH* in tissues at two developmental stages (abaxial lip, adaxial lip, petal, and sepal from the bud and flower). We normalized *CHS* and *BCH* expression with the most stable and most unstable reference genes according to geNorm and NormFinder. qRT-PCR experimental method was the same as described above, and the relative expression level was calculated by 2^−ΔΔct^ method [55]. Each include three technical replicates. The FPKM values of *CHS* and *BCH* in transcriptome data can be used as a reference for their expression patterns.

## Figures and Tables

**Table 1:** Selected candidate reference genes, primers, and amplification characteristics.

Gene Name	Description	Primer Sequence (5′-3′)	Amplicon Length (bp)	RT-qPCR Efficiency (%)	R^2^
*ACT7*	actin-7-like	F:AACTGGTATTGTGCTGGATTC R:TCATCAGTGAATCTGTAAGGTC	128	88.58	0.9970
*ACTF11*	actin-depolymerizing factor 11-like	F:ATGTTCATCAACAGTTGCAG R:GGCAGTGATCATCAACTC	130	158.30	0.8844
*ATP*	ATP synthase subunit O	F:TTCACTGATCAATTACGGC R:GATGCGTAGTTTCCAGTAC	141	102.44	0.9496
*EF1* δ	elongation factor 1-delta	F:CTACCAAGCTTCAAAGGATG R:CTCAGATACAGTAGTAGACC	143	98.32	0.9984
*MADH*	malate dehydrogenase	F:CTACGATATCGCTGGTACTC R:ACGAGTTCTGATCCCTCC	123	105.62	0.9948
*g-TUB*	gamma-tubulin complex component 2	F:ATCCATTGTGATTGAGAAGGC R:ACTGTAGTATCACCTGCCATG	102	93.05	0.9996
*UBC*	Ubiquitin-conjugating enzyme	F:ATCTCTCAGGCAAGCATTAC R:GTAGAGGTATGGCACTAATC	125	130.87	0.9856
*26S*	26S proteasome non-ATPase regulatory subunit 2	F:CAGAAGCTCGCACTAGAG R:TATGGGCAGATCATCATACTG	162	102.60	0.9953
*40S*	40S ribosomal protein	F:GAAGATGGTATTCCTGCAG R:TAGCCTTGGCTGCTTCATG	139	93.18	0.9999
*60S*	60S ribosomal protein	F:GTCCAAGTCGAATCAGTATG R:ATAGTGCGTGCCATTCTTC	139	96.68	0.9960

**Table 2:** Expression stability of the nine candidate reference genes as calculated by NormFinder.

Sample	Rank	1	2	3	4	5	6	7	8	9	10
Total	Gene	*EF1δ*	*60S*	*40S*	*ACT7*	*ATP*	*g-TUB*	*ACTF11*	*UBC*	*MADH*	*26S*
	stability	0.068	0.074	0.124	0.188	0.255	0.332	0.427	0.494	0.645	2.383
Bud	Gene	*60S*	*EF1δ*	*ATP*	*40S*	*g-TUB*	*ACT7*	*ACTF11*	*UBC*	*26S*	*MADH*
	stability	0.143	0.166	0.193	0.227	0.287	0.333	0.360	0.368	0.385	0.388
Flower	Gene	*ATP*	*60S*	*EF1δ*	*ACTF11*	*ACT7*	*g-TUB*	*40S*	*MADH*	*UBC*	*26S*
	stability	0.020	0.020	0.052	0.084	0.104	0.156	0.279	0.341	0.437	2.615
Bud and Flower	Gene	*ACT7*	*60S*	*EF1δ*	*40S*	*ATP*	*g-TUB*	*UBC*	*ACTF11*	*MADH*	*26S*
	stability	0.004	0.004	0.034	0.081	0.280	0.303	0.364	0.465	0.623	1.519
Abaxial lip	Gene	*ATP*	*40S*	*60S*	*EF1δ*	*UBC*	*ACT7*	*g-TUB*	*MADH*	*ACTF11*	*26S*
	stability	0.004	0.004	0.011	0.026	0.026	0.043	0.356	0.658	0.962	4.283
Adaxial lip	Gene	*ACTF11*	*g-TUB*	*60S*	*EF1δ*	*40S*	*ACT7*	*ATP*	*MADH*	*UBC*	*26S*
	stability	0.012	0.012	0.124	0.165	0.192	0.289	0.558	0.766	0.822	2.476
Petal	Gene	*ACT7*	*60S*	*EF1δ*	*40S*	*ATP*	*ACTF11*	*g-TUB*	*UBC*	*MADH*	*26S*
	stability	0.049	0.049	0.062	0.245	0.383	0.393	0.454	0.645	0.654	1.053
Sepal	Gene	*ACT7*	*60S*	*EF1δ*	*ATP*	*40S*	*g-TUB*	*ACTF11*	*UBC*	*MADH*	*26S*
	stability	0.041	0.087	0.163	0.191	0.343	0.389	0.561	0.676	0.756	0.844

## Supplementary Materials

The following are available online at: https://www.mdpi.com/article/10.3390/ijms23020738/s1.

## Abbreviations

qRT-PCR	Quantitative real-time polymerase chain reaction
Cq	Cycle threshold
*ACT7*	Actin-7-like
*ACTF11*	Actin-depolymerizing factor 11-like
*ATP*	ATP synthase subunit O
*EF1* δ	Elongation factor 1-delta
*MADH*	Malate dehydrogenase
*g-TUB*	gamma-tubulin complex component 2
*UBC*	Ubiquitin-conjugating enzyme
*26S*	26S proteasome non-ATPase regulatory subunit 2
*40S*	40S ribosomal protein
*60S*	60S ribosomal protein
ABP	Anthocyanins biosynthetic pathways
CBP	Carotenoids biosynthetic pathways
*CHS*	Chalcone synthase
*BCH*	β-carotene hydroxylase
FPKM	Fragments per Kilobase of transcript per millions base pairs sequenced
