# Supplementary material for: Selection and Validation of Reference Genes for Quantitative Real-Time PCR Analysis of Development and Tissue-Dependent Flower Color Formation in Cymbidium lowianum
Source: Int J Mol Sci. 2022 Jan 10;23(2):738. doi: 10.3390/ijms23020738 (PMC8776083; doi:10.3390/ijms23020738)
Supplement: Supplementary file 1 [file ijms-23-00738-s001.zip › ijms-1455484-supplementary.pdf]

Supplementary data

Figure S1. Detection of amplicon size and primer specificity of the selected reference genes. Agarose gel showed specific RT-PCR products of the detected genes amplified from mix of cDNA (cDNA of abaxial lip, adaxial lip, petal, and sepal from bud and flower were equally mixed).

Figure S2. Melting curves for the ten candidate reference genes. Mixture of cDNA (cDNA of abaxial lip, adaxial lip, petal, and sepal from bud and flower were equally mixed), (1, 1/5, 1/25, 1/125, 1/625) of the mix was used for the test. Each dilution included three technical replicates.

Figure S3. Standard curves of ten candidate reference genes.

Table S1. FPKM values of selected reference genes

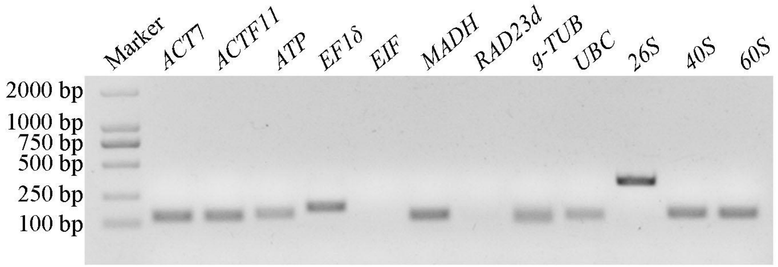

Figure S1. Detection of amplicon size and primer specificity of the selected reference genes. Agarose gel showed specific RT-PCR products of the detected genes amplified from mix of cDNA (cDNA of abaxial lip, paraxial lip, petal, and sepal from bud and flower were equally mixed).

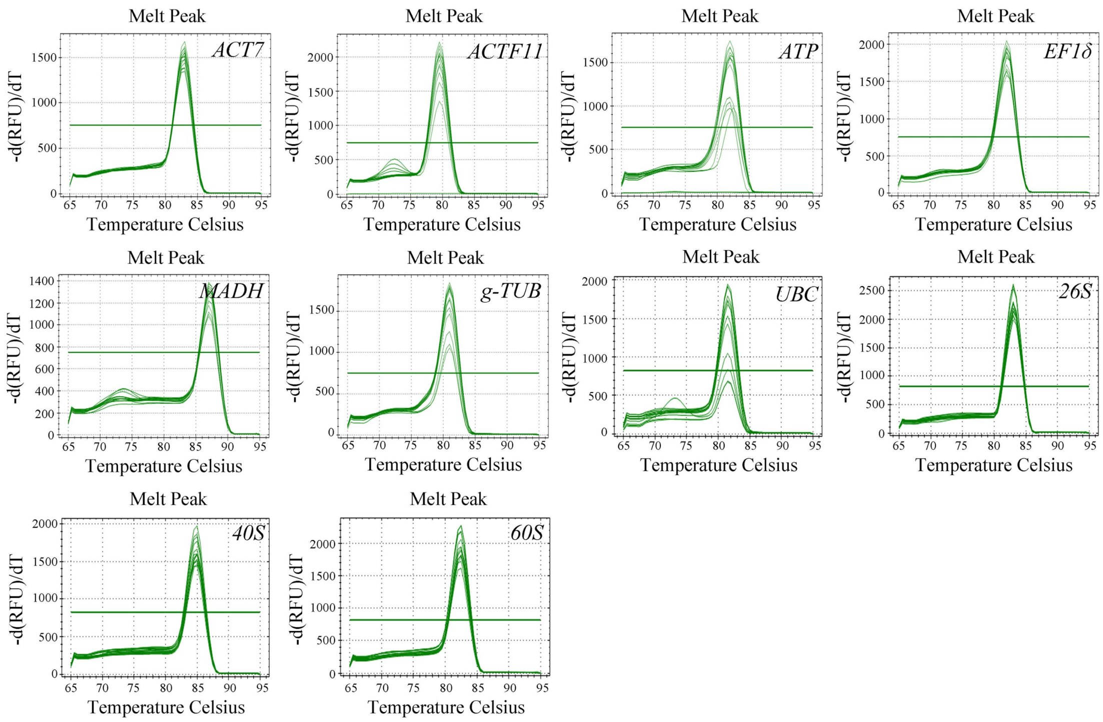

Figure S2. Melting curves for the ten candidate reference genes. Mixture of cDNA (cDNA of abaxial lip, paraxial lip, petal, and sepal from bud and flower were equally mixed), (1, 1/5, 1/25, 1/125, 1/625) of the mix was used for the test. Each dilution included three technical replicates.

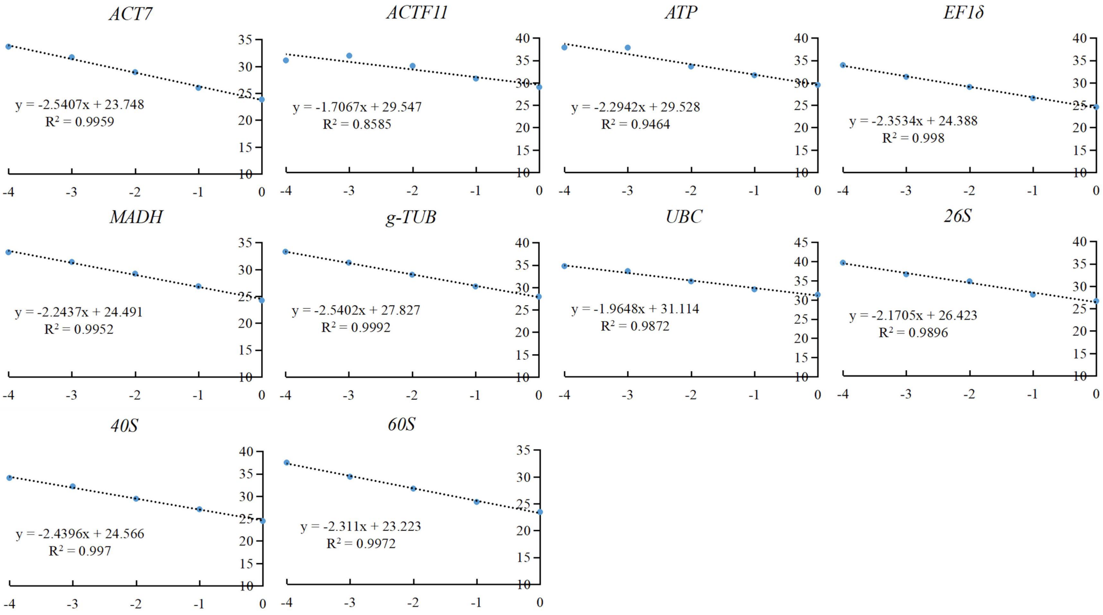

Figure S3. Standard curves of ten candidate reference genes.

Table S1. FPKM values of selected reference genes

Gene \ Sample	Bud				Flower			
	**Abaxial lip**	**Adaxial lip**	**Petal**	**Sepal**	**Abaxial lip**	**Adaxial lip**	**Petal**	**Sepal**
ACT7	912.94	818.60	868.35	908.72	755.68	862.89	721.39	774.79
ACTF11	195.85	224.87	201.43	188.84	164.93	171.85	219.81	203.76
ATP	111.99	123.79	117.91	98.99	119.38	105.43	110.68	115.51
EF1δ	190.04	231.30	230.64	188.76	204.41	223.79	197.50	208.04
MADH	100.30	119.08	102.49	90.69	107.74	115.43	117.47	113.55
g-TUB	11.52	14.92	13.29	12.63	6.83	8.52	9.19	9.43
UBC	192.79	193.27	172.89	157.21	198.03	219.43	198.26	202.87
26S	117.90	129.31	121.35	123.96	89.09	117.27	137.01	124.74
40S	163.55	191.59	193.86	164.21	170.96	159.79	136.09	158.09
60S	111.84	123.60	140.5	109.98	145.33	137.37	129.14	144.76
